# Measuring Social Exclusion in Routine Public Health Surveys: Construction of a Multidimensional Instrument

**DOI:** 10.1371/journal.pone.0098680

**Published:** 2014-05-30

**Authors:** Addi P. L. van Bergen, Stella J. M. Hoff, Erik J. C. van Ameijden, Albert M. van Hemert

**Affiliations:** 1 Municipal Health Service Utrecht, Utrecht, The Netherlands; 2 The Netherlands Institute of Social Research|SCP, The Hague, The Netherlands; 3 Leiden University Medical Center, Department of Psychiatry, Leiden, The Netherlands; University of St Andrews, United Kingdom

## Abstract

**Introduction:**

Social exclusion is considered a major factor in the causation and maintenance of health inequalities, but its measurement in health research is still in its infancy. In the Netherlands the Institute for Social Research (SCP) developed an instrument to measure the multidimensional concept of social exclusion in social and economic policy research. Here, we present a method to construct a similar measure of social exclusion using available data from public health surveys.

**Methods:**

Analyses were performed on data from the health questionnaires that were completed by 20,877 adults in the four largest cities in the Netherlands. From each of the four questionnaires we selected the items that corresponded to those of the SCP-instrument. These were entered into a nonlinear canonical correlation analysis. The measurement properties of the resulting indices and dimension scales were assessed and compared to the SCP-instrument.

**Results:**

The internal consistency of the indices and most of the dimension scales were adequate and the internal structure of the indices was as expected. Both generalisabiliy and construct validity were good: in all datasets strong associations were found between the index and a number of known risk factors of social exclusion. A limitation of content validity was that the dimension “lack of normative integration” could not be measured, because no relevant items were available.

**Conclusions:**

Our findings indicate that a measure for social exclusion can be constructed with available health questionnaires. This provides opportunities for application in public health surveillance systems in the Netherlands and elsewhere in the world.

## Introduction

Social exclusion is generally considered as one of the social determinants of health and a major factor in the causation and maintenance health inequalities [1]–[3]. Social exclusion is a broad term that refers to the inability of certain groups or individuals to participate fully in society. The World Health Organization defines social exclusion as “dynamic multidimensional processes driven by unequal power relationships interacting across four main dimensions - economic, political, social and cultural - and at different levels including individual, household, group, community, country and global levels” [4]. Important features of social exclusion are multi-dimensionality, relativity (i.e. social exclusion is context specific) and agency [5]. Agency refers to the fact that the excluding is done by someone or something, which can be the government or private institutions, the social environment or the individual itself. It is common that exclusion processes in one dimension affect those in other dimensions [2], [6], [7]. For example the loss of paid employment may lead to loss of social contacts and loss of income, which in turn may result in debts, poor housing, insecure living environment or reduced access to health care [6]. All these factors increase the risk of health problems directly or indirectly. In addition the experience of being excluded affects health negatively [1], [2]. Health risks thus tend to accumulate in socially excluded individuals and groups.

In the Netherlands, Community Health Services are responsible for public health monitoring at the local level. At least once every four years they conduct routine public health surveys among the adult population. The questionnaires that are used for this cover a broad spectrum of health outcomes and determinants. In addition to mandatory questions on a national level, topics can be included to address local policy priorities. If available, validated and standardised measures are used [8]. Measurement of social in these health surveys is desired, but acceptable measurement instruments are lacking.

Recently, the Netherlands Institute for Social Research|SCP (SCP) has developed an instrument to measure social exclusion in social and economic policy research [9], [10]. Based on an extensive literature review, the SCP has first defined and then operationalised the concept of social exclusion [7]. The definition is rooted in two scientific traditions i.e. the French tradition, which focuses on the extent to which people are integrated into society and connected to others (socio-cultural exclusion); and the Anglo-Saxon tradition, which emphasises relative deprivation, the notion that people or groups consider themselves disadvantaged compared to others with similar characteristics (their reference group). Nowadays, research within the Anglo-Saxon tradition is focused on a more ‘objective’ approach in terms of social indicators that measure differences in socio-economic status and rights (structural-economic exclusion). [9].

The SCP definition of social exclusion distinguishes two forms of social-cultural exclusion: “lack of normative integration” and “limited social participation” and two forms of structural-economic exclusion i.e. “material deprivation” and “inadequate access to basic social rights”. A person is socially excluded to some extent if there is accumulation of deficiencies on or more of these four dimensions. The greater the number of deficiencies and the larger these deficiencies are, the higher the degree of social exclusion. See Table 1 for the operationalisation of the dimensions. To construct an instrument to measure the four dimensions, the SCP administered a questionnaire to a sample of the Dutch population. The initial questionnaire consisted of 232 items derived from previous SCP research, literature, focus groups and cognitive tests. For each of the dimensions, a subscale containing three to four items was constructed by using nonlinear canonical correlation analysis. Together, these 15 items make a general index that reflects the underlying construct of social exclusion. The general index measures the degree of social exclusion at the individual level, with a higher index score for persons deprived simultaneously on several dimensions. [9], [10].

**Table 1:** Operationalisation of the four dimensions of social exclusion. [9], [10].

Dimension of social exclusion	Operationalisation
*Lack of normative integration*	Non-compliance with core values of society. In the Dutch context,this relates to issues like “having no respect for other people”,“not saying ‘thank you’ when receiving change”or “putting out your garbage on a Tuesday when it’sonly allowed on a Wednesday…..”*.
*Limited social participation*	Social isolation, limited participation insocial networks and inadequate social involvement.
*Material deprivation*	Deficits that people experience as shown by debts andthe absence of certain basic goods and services, such as awashing machine or a daily hot meal.
*Inadequate access to basic social rights*	Inability to exercise the rights people normally have.This dimension is operationalised as having access toadequate health care, sufficient education and a proper living environment.

*The quotations are from participants in the focus groups organised by the SCP [10]

Although the SCP measurement instrument for social exclusion has been adapted and validated for the Dutch context, its suitability for routine public health surveys is limited. The Community Health Services consider the measure, with 15 items, too long to include in their health questionnaires. The total number of items that can be included in the questionnaires is limited and there is fierce competition between topics. Moreover, there is substantial overlap of the SCP-questionnaire of Social Exclusion with current topics of the health surveys, such as loneliness, social capital, financial situation and housing. This last observation prompted us to explore whether the multidimensional concept of social exclusion can validly be approximated with items from the health questionnaires that are already used in the public health surveys in the Netherlands. We had access to the data collected in the surveys of 2008 with health questionnaires from the Community Health Services of the four largest cities in the Netherlands. Our ultimate goal is to develop a nationally validated and standardised measure to monitor social exclusion in routine public health surveys.

## Methods

### Ethics Statement

Ethical approval was not required as this study relied on secondary anonymised data collected in the context of performing statutory tasks (Public Health Act of the Netherlands), in strict accordance with the national standard. At no point in time did the datasets contain direct identifiers. Codes to track response were removed from paper questionnaires directly upon receipt and processed separately, as were online access codes. The risk of re-identification of individuals from indirect identifiers such as age (in years) and sex, was very low.

The datasets are freely available for non-commercial research purposes.

### Data Source and Participants

We conducted secondary analysis on data of four public health surveys that were collected in 2008 by the local Community Health Services in the cities of Amsterdam, Rotterdam, The Hague and Utrecht, using a uniform research methodology. The content of the questionnaires was only dissimilar for items that were selected according to local policy priorities.

In each city an a select sample was drawn from the non-institutionalised population aged 16 years and older, stratified by district, neighbourhood, age and ethnicity. A total of 42,686 persons received a questionnaire by mail. These questionnaires could be filled out in writing or via the Internet. Non-responders received a reminder after two weeks. In addition, difficult to reach groups such as non-western immigrants and residents of deprived neighbourhoods were contacted after four weeks by telephone or home visit and invited to participate by mail or personal interview in the language preferred by the respondent. For Turkish respondents, the main non-Dutch speaking minority in the Netherlands, a translated questionnaire was available.

The overall response rate was 50% (20,877 respondents) and ranged between 47% in Rotterdam and 54% in Utrecht. Despite the intensive follow-up, the response was lower among difficult to reach groups. Through oversampling these groups were still well represented in each of the four studies [11]. In line with the age standard for public health surveys in the Netherlands, we limited our analyses to respondents aged 19 years and older (19,658 respondents).

### Construction of Measurement Instrument

**Figure 1:**
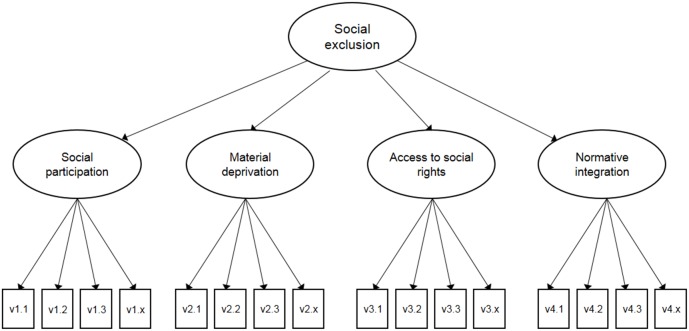
Measuement model for social exclusion. The model illustrates the construction of a composite index based on selected sets of variables, that each measures one of the four dimensions of social exclusion.

Following the SCP procedures, we applied non-linear canonical correlation analysis (OVERALS) to the different sets of survey data. OVERALS is an optimal scaling technique developed by the University of Leiden, which is available in the SPSS software package. Canonical correlation analysis is often used to explore relationships between two sets of variables, an independent and dependent set, and to reduce the dimensionality to a few linear combinations of the measures under study [13]. In the context of the current study, we used canonical correlation analysis to construct a composite index based on selected sets of variables, each measuring one of the four dimensions of social exclusion (Figure 1). OVERALS differs in three ways from standard linear canonical correlation analysis: variables can be nominal, ordinal or interval; there can be more than two sets of variables; and instead of maximizing correlations between the variable sets, the sets are compared to an unknown compromise set that is defined by the object scores [13]. If the correlation between the sets is sufficient, it is assumed that these sets refer to an underlying concept. [9], [12].

From each dataset we selected items matching one of the four dimensions of social exclusion as operationalised by the SCP. All items were coded in the same direction, so that a high score refers to more exclusion. Records with one or more missing values on all dimensions were removed from the analyses. As the items in The Hague and Rotterdam datasets matched exactly, these were merged. The analysis thus resulted in three indices: Amsterdam (Index1), Rotterdam/The Hague (Index2) and Utrecht (Index3).

Initially all items were entered in the OVERALS analysis. Using category quantifications, the most appropriate measurement level of the items was chosen. Similar to the SCP method [9], [12], items with component loadings less than 0.300 were removed one by one, starting with lowest correlations. Subsequently, items with weights less than 0.100 were removed, as well as items that scored in the opposite direction. Finally, scores on the subscales were computed using category quantifications and weights (for formulas see [12]).

### Measurement Properties

We used a series of methods to evaluate the measurement properties of the constructed indices, i.e. content validity, internal consistency, internal structure and construct validity.

To assess the content validity, we examined whether the constructed indices encompassed all dimensions of social exclusion and whether the included items were representative for the dimensions they were expected to measure. In addition, we inspected the distributions of the index scores and compared these with the SCP index. To assess the internal consistency of the indices we calculated the canonical correlation, which measures the degree to which the items contribute to the underlying latent variable. A canonical correlation of 0.300 was defined as the lower limit to ensure reliability of the indices [9], [13]. Cronbach’s alpha was used to measure the internal consistency of the subscales, where we considered α≥0.70 to be good [14].

For the assessment of the internal structure of the instruments, we computed the intercorrelations of the subscales and the general indices. Construct validity was assessed by testing predefined hypotheses [14]. For this purpose we selected a number of items that measure risk factors and correlates of social exclusion, derived from previous SCP research [7], [10]. None of these were selected for the construction of the indices. The factors and correlates included were:

*Sociodemographic variables*: low educational level; non-Western ethnic background; single-parent; living alone; unemployed and/or recipient of social security or disability benefits; no paid job; income below modal (1,700 Euros net per month); and living in a deprived neighbourhood;
*Health related factors*: fair or poor self-rated health (versus good or very good); being diagnosed with at least one of eighteen chronic conditions; impaired in daily activities at home, at school, at work or in their leisure time due to chronic conditions (light to strong) and high risk for anxiety and depression disorder (score 30 or higher on Kessler psychological distress scale);
*Variables on self-reliance*: low perceived life control (Pearlin & Schooler Mastery Scale, score < = 19); and need of help to complete the health questionnaire.

We expected higher levels of social exclusion in these groups. The construct validity was considered satisfactory if at least 75% of the associations were in correspondence with these expectations [14].

### Index Selection

Based on the results of the measurement properties analyses, we identified the best performing index. Generalisability of this index was subsequently examined by testing the items in the other datasets, where available.

### Statistical Analysis

Analyses were performed with SPSS 19.0. Group differences were tested with Pearson Chi Square test (categorical variables) or Anova F-test (continuous variables). Linear regression analyses were used to assess relationships between risk factors and social exclusion indices.

## Results

### Characteristics of the Study Populations

Table 2 shows the sociodemographic characteristics of respondents in the four cities. As can be seen in Table 2, risk groups for social exclusion such as persons of non-Western origin, lower educated persons and persons living in deprived neighbourhoods were well represented in all four samples. Significant differences were found between the samples with regard to sex, age, ethnical background, educational level and the proportion of individuals living in deprived neighbourhoods. The observed differences reflect demographic variation between the four cities and the degree of oversampling in difficult to reach groups.

**Table 2:** Sociodemographic characteristics of the respondents per sample (unweighted).

	Total	Amsterdamsample	Rotterdamsample	The Haguesample	Utrechtsample	p
	(N = 19,658)	(N = 6,511)	(N = 5,127)	(N = 4,220)	(N = 3,800)	
Sex (male, %)	43.3	41.1	45.8	44.2	42.5	.000*
Age (mean, SD)	51.0 (19.1)	58.2 (20.0)	49.3 (17.6)	48.8 (17.6)	43.3 (16.9)	.000&
Non-Western ethnicbackground (%)	20.4	19.1	23.4	24.8	13.7	.000*
Low educational level (%) #	16.1	19.8	15.8	14.8	11.5	.000*
Living in a deprived neighbourhood (%)	34.2	39.6	30.7	36.2	27.2	.000*

*The P values were obtained by using Pearson’s Chi Square analysis.

& The P value was obtained by using One-way Anova F-test.

#No education and primary school.

### Construction of the Measurement Instruments

In the four health questionnaires, we identified 11 items that matched the SCP operationalisation of the dimension limited social participation. All of these items belong to the loneliness scale of De Jong Gierveld [15]. In addition, in the Utrecht questionnaire 3 items were available on the frequency of social contacts.

For the measurement of the dimension ‘material deprivation’ 2 items were available in each of the cities. These items relate to the financial situation of the household and difficulties in making end meets.

We found no items to measure ‘inadequate access to basic social rights’ in the Amsterdam sample. In the questionnaires of The Hague, Rotterdam and Utrecht we found 18 items that matched the operationalisation of this dimension by the SCP, including 5 items on neighbourhood cohesion, 2 items on satisfaction with housing and living environment and 11 items on environmental and nuisance problems in the neighbourhood. The questionnaires from The Hague and Rotterdam included 2 additional items on feeling unsafe during the day or night. From the Utrecht questionnaire 26 additional items were selected that related to the presence of moisture or mold in the home, to the need for information or assistance with health problems and to the need for facilities in the neighbourhood.

Items for the dimension ‘lack of normative integration’ were not available in any of the questionnaires.

With the aforementioned 62 items, three indices were constructed: Index1 was based on the items from the Amsterdam questionnaire, Index2 on the items from the Rotterdam and The Hague questionnaires combined and Index3 on the items from the Utrecht questionnaire. In Index1, 8 of the 13 items were retained, in Index2 14 of the 33 items and in Index3 17 of the 57 items. With one exception, items were removed because of low component loadings or low weights. The item on ‘mold and moisture in the home’ from the Utrecht questionnaire was removed because of a reverse association with the other items. The centroid plots generated by the OVERAs001LS analyses are given in Figures S1, S2 and S3.

Table 3 shows the selected items per index and per dimension. From the 14 items that were present in two or more datasets, 10 were included in all relevant indices and 4 items were included in some indices but not in others. For example, the item ‘There are enough people I feel close to’ was incorporated in the indices 2 and 3 but not in Index3. Instead, Index3 contained the item ‘I miss having people around’, which was absent in the indices 1 and 2.

**Table 3:** Summary of items which were incorporated in the SCP index or in one of the three constructed indices, by dimension and index.

Dimension	Items SCP	Items healthquestionnaires	Index1(Amsterdam)*	Index2(Rotterdam/TheHague)*	Index3(Utrecht)*
*Dimension 1:* *‘Limited social* *participation’*	There are peoplewho genuinely understand me	There are enough peopleI feel close to	+	+	-
	I feel cut off from other people	I experience a generalsense of emptiness	+	+	+
	There are people with whomI can have a good conversation	There is always someoneI can talk to about myday-to-day problems	+	-	+
	I have contactwith neighbours	Little contact with neighboursand people in the street	.	.	+
		There are plenty of peopleI can lean on when I haveproblems	+	+	+
		I miss the pleasure of thecompany of others	+	+	+
		I often feel rejected	+	+	+
		I miss having people around	-	-	+
*Dimension 2:* *‘Material* *deprivation’*	I have enough money to heatmy home		.	.	.
	I have enough money for clubmemberships		.	.	.
	I have enough money to visitothers		.	.	.
	I have enough money to meetunexpected expenses		.	.	.
		Had difficulty past yeargetting by on thehousehold income	+	+	+
		Current financial situation ofthe household: haveto go into debt	+	+	+
*Dimension 3:* *‘Inadequate* *access to basic* *social rights’*	We all get on well in ourneighbourhood	People in this neighbourhoodgenerally do not getalong with each other	.	+	+
	I am satisfied withthe quality of my home	Degree of satisfactionwith housing	.	+	+
	I didn’t receive a medicalor dental treatment		.	.	.
		The people in my neighbourhoodhelp each other	.	+	-
		People in this neighbourhoodcan be trusted	.	+	+
		I prefer not to socialise withpeople in my neighbourhood	.	+	+
		Feeling unsafe during the day	.	+	.
		Feeling unsafe in theevening and at night	.	+	.
		Need for informationor assistance: stress reduction	.	.	+
		Need for informationor assistance: copingwith depression	.	.	+
		Need for informationor assistance: coping withloneliness	.	.	+
		Need for informationcentre on care and welfare	.	.	+
*Dimension 4:* *‘Lack of* *normative* *integration’*	I give to good causes (no)		.	.	.
	I sometimes dosomething for myneighbours (no)		.	.	.
	I put glass items inthe bottle bank (never)		.	.	.
	Work is just a way ofearning money (agree)		.	.	.

The table lists the items that became included in one of the three constructed indices as well as the items that form part of the SCP index. The SCP index is is shown for reference purposes only. Per index the following information is displayed:

* + retained in OVERALS analysis; − removed in OVERALS analysis; item not available in respective dataset.

### Measurement Properties

#### Content validity

To examine the degree to which the indices cover the multidimensional concept of social exclusion, we compared, for each dimension, the items in the constructed indices with those in the SCP index. The dimension ‘limited social participation’ of the SCP index comprises items on social isolation and on participation in social networks. From Table 3 we can see that all three constructed indices included items on social isolation, but only Index3 contained an item on participation in social networks i.e. contacts with neighbours. In the dimension ‘material deprivation’ the SCP index includes items on the financial situation of the household and on the lack of basic goods and services. The three constructed indices did contain 2 items on the financial situation of the household, but items on the lack of basic goods and services, were absent in all three indices. In the dimension ‘inadequate access to basic social rights’ the SCP index contains aspects of good living environment and access to health care. Index2 and Index3 contained similar items on good living environment, but only Index3 contained additional items on access to healthcare. These items however, referred to the need for information or assistance and not the actual lack of access, as does the SCP questionnaire.

As floor or ceiling effects may limit the content validity [14], we examined the frequency distributions of the three indices. All three distributions were right-skewed, which corresponds well with the distribution of the SCP index and is consistent with the expectation that a large part of the population is not excluded, while the degree of exclusion at the right end of the scales varies widely.

#### Internal consistency

Table 4 shows the findings on the internal consistency of the indices and subscales. The canonical correlations of three constructed indices ranged from 0.35 (Index1) to 0.44 (Index3), which is sufficient. Index2 and Index3 had even higher canonical correlations than the SCP index (r_d_ = 0.38). The Cronbach’s alphas of the transformed subscales were good for dimensions 1 and 2. For dimension 3 Cronbach’s alphas were 0.68 (Index2) and 0.65 (Index3). In the SCP study Cronbach’s alphas were not calculated.

**Table 4:** Canonical correlation analyses summary table for the three constructed indices: component loadings and weights per item, Crohnbach’s alpha per subscale and canonical correlation per index.

	Index1Amsterdam			Index2The Hague & Rotterdam			Index3Utrecht		
	(n = 6,368)			(n = 9,238)			(n = 3,763)		
	Componentloading*^a^*	Weight*^b^*	Cronbach’salpha	Componentloading*^a^*	Weight*^b^*	Cronbach’salpha	Componentloading*^a^*	Weight*^b^*	Cronbach’salpha
*Set 1: ‘Limited social participation’*			0.81			0.80			0.79
There are enough people I feel close to (rev)	0.50	0.11		0.52	0.16				
I experience a general sense of emptiness	0.64	0.20		0.62	0.17		0.61	0.20	
There is always someone I can talk to about my day-to-day problems (rev)	0.50	0.13					0.57	0.20	
Little contact with neighbours and people in the street							0.32	0.16	
There are plenty of people I can lean on when I have problems (rev)	0.57	0.19		0.55	0.20		0.56	0.20	
I miss the pleasure of the company of others	0.63	0.20		0.64	0.21		0.63	0.14	
I often feel rejected	0.66	0.30		0.68	0.33		0.66	0.25	
I miss having people around							0.62	0.13	
*Set 2: ‘Material* *deprivation’*			0.72			0.75			0.71
Had difficulty past year getting by on the household income	0.80	0.66		0.73	0.54		0.68	0.56	
Current financial situation of the household:have to go into debt	0.63	0.26		0.63	0.30		0.54	0.23	
*Set 3: ‘Inadequate access to basic social rights’*						0.68			0.65
The people in my neighbourhood help each other (rev)				0.35	0.12				
People in this neighbourhood can be trusted (rev)				0.44	0.13		0.36	0.11	
People in this neighbourhood generally do not get along with each other				0.39	0.14		0.36	0.15	
I prefer not to socialise with people in my neighbourhood				0.38	0.13		0.33	0.10	
Degree of satisfaction with housing				0.57	0.39		0.48	0.29	
Feeling unsafe during the day				0.44	0.18				
Feeling unsafe in the evening and at night				0.42	0.14				
Need for information or assistance: stress reduction							0.47	0.19	
Need for information or assistance: coping with depression							0.50	0.12	
Need for information or assistance: coping with loneliness							0.57	0.34	
Need for information centre on care and welfare							0.34	0.16	
*Canonical correlation of the general index* c d			0.35			0.40			0.44

Analyses were performed with SPSS OVERALS module. OVERALS calculates a.o. component loadings, weights and eigenvalues.

a b Component loadings in OVERALS are similar to factor loadings in a factor analysis. Weights are similar to standardised regression coefficients [9], [12].

c The canonical correlation is calculated with the formula: r_d_ = ((K x E_d_)–1)/(K–1), whereby K = number of sets, d = factor number (in this case only one factor was calculated), and E = the eigenvalue of the factor/index.

d SCP index: canonical correlation = 0.38 [10].

#### Internal structure

Table 5 provides the correlations between the subscales and the general indices and between the subscales themselves. The correlations between the subscales and the general indices ranged from 0.68 to 0.82, and were similar to those of the SCP index. As expected, the correlations between the subscales were weaker than with the general indices. They ranged from 0.33–0.55, which is in line with the internal structure of the SCP index.

**Table 5:** Pearson correlations coefficients between general indices and dimension subscales, SCP and the four cities.

Correlation between:	SCPIndex^a^	Index1Amsterdam	Index2Rotterdam & TheHague	Index3Utrecht
General index x dimension 1	0.76	0.78*	0.76*	0.82*
General index x dimension 2	0.70	0.79*	0.72*	0.68*
General index x dimension 3	0.77		0.73*	0.81*
Dimension 1 x dimension 2	0.35	0.30*	0.34*	0.33*
Dimension 1 x dimension 3	0.43		0.39*	0.55*
Dimension 2 x dimension 3	0.44		0.34*	0.38*

*p<.01.

a Vrooman and Hoff [10].

#### Construct validity

As can be seen from Table 6 all predefined hypotheses were confirmed. Without exception, the indices were positively associated with the selected risk factors and correlates. Regression coefficients showed the expected direction and were statistically significant (p<0.01). Persons with lower income were more often socially excluded than people with a higher income. People in poor health, persons of non-Western origin and those with low perceived self control were also at higher risk. The same holds for lower educated persons, people living in deprived neighbourhoods, jobless adults, single persons and single parents. In contrast to the SCP, we also found significant associations with low labour market position and need of assistance in filling in the questionnaire. In general, the associations found in the current research were stronger than in the SCP study.

**Table 6:** Association between social exclusion indices and known risk factors and correlates: standardised regression coefficients and p-values.

		Index1Amsterdam		Index2Den Haag enRotterdam		Index3Utrecht		IndexSCP^a^	
		(n = 6368)		(n = 9238)		(n = 3763)		(n = 574)	
		β	p	β	p	β	p	β	p
Educational level	Low educational level (no education and primary school)	0.22	**	0.24	**	0.24	**	0.12	**
Ethnic background	Non-Western ethnic background	0.28	**	0.34	**	0.33	**	0.18	**
Family situation	Single parent with underage child(ren)	0.11	**	0.13	**	0.10	**	0.13	**
	Living alone	0.14	**	0.13	**	0.15	**	0.16	**
Labour marketposition (54 years oryounger)	Unemployed and/or recipient of social securityor disability benefits. (SCP:Receives unemployment benefit,disability benefit or social assistance benefit)	0.34	**	0.36	**	0.32	**	-0.03	ns
	No paid job	0.19	**	0.19	**	0.23	**	0.02	ns
Income	Income below modal (1,700 Euros net per month)	0.41	**	0.41	**	0.38	**	0.23	**
Health	Self-rated health fair or poor	0.34	**	0.38	**	0.37	**	0.19	**
	Diagnosed with at least one chronic condition.(SCP: Suffers from a disability or a chronic condition)	0.19	**	0.19	**	0.18	**	0.09	*
	Impaired in daily activities at home,at school, at work or in theirleisure time owing to chronic conditions	0.29	**	0.29	**	0.25	**		
	High risk for anxiety and depressiondisorder. (SCP: Low subjective well-being)	0.43	**	0.45	**	0.43	**	0.30	**
Self-reliance	Received help in completing thehealth questionnaire.(SCP: Needs help filling in forms)	0.16	**	0.16	**	0.18	**	0.06	ns
	Low perceived life control &			0.31	**	0.46	**		
Neighbourhood	Living in deprived neighbourhood	0.15	**	0.24	**	0.24	**		

*Significant effect, p<0.05;

**Significant effect, p<0.01,

ns Not significant, p> = 0.05.

% Kessler psychological distress scale (K10), score 30 or higher.

& Pearlin & Schooler Mastery Scale, score < = 19.

a Vrooman and Hoff [10].

Explanatory note. Linear regression analyses were used to assess relationships between social exclusion indices and known risk factors and correlates. Construct validity was considered satisfactory if at least 75% of the associations were in correspondence with predefined hypotheses.

### Index Selection and Generalisabilty

When compared with the other indices, Index3 performed best on content validity and performed equally well with regard to internal consistency, internal structure and construct validity. For that reason we continued our analysis with Index3. Generalisability of the items from Index3 was tested in the datasets of Rotterdam/The Hague and Amsterdam, where available. We performed analyses with 2 and 3 sets of variables. In all cases, the OVERALS analysis yielded indices with comparable measurement properties i.e. a distribution of index scores, internal validity, internal structure and construct validity that was similar to Index3.

## Discussion and Conclusions

Our approach to construct a scale for social exclusion based on items from routine public health surveys was successful in all four cities as far as relevant items were available in the surveys. Data reduction with canonical correlation analysis yielded fairly similar selections of items consistently with the original SCP index. This corroborates the assumption that similar constructs were measured. Both the general indices and the underlying dimension scales had good internal consistencies, with the exception of the dimension scale ‘inadequate access to basic social rights’. In line with the SCP index, the internal structure of our indices reflected the multidimensional character of the concept social exclusion. Moreover, the indices demonstrated strong associations with risk factors and correlates, which may be considered as a confirmation of the construct validity of the indices. On the whole, Index3, based on the Utrecht dataset, performed most consistent due to better content validity in the dimensions ‘limited social participation’ and ‘inadequate access to basic social rights’. The OVERALS analyses demonstrated good generalisability to the other cities.

The measurement of a multidimensional construct such as social exclusion provides methodological challenges for researchers [3]. By using nonlinear canonical correlation analysis (OVERALS), we were able to construct an instrument which not only expressed the degree of social exclusion in a single index score, but also allowed us to simultaneously assess the underlying dimensions. The selection of items and attribution of weight factors were not defined a priori but were determined empirically by the OVERALS technique, and thus added credibility to our findings.

In our research, we had the advantage of an available well-defined and established definition and operationalisation of social exclusion used in social and economic policy research. The definition, was based on extensive literature and empirical research [7], [10], [16], and was validated by the SCP in various Dutch population groups such as the elderly and children [17]–[19], and in several European countries [20].

Due to the number and redundancy of items, the measurement instrument developed by the SCP [10], however, is difficult to accommodate in routine pubic health monitoring. Undue length and overlap with available questions may lead to lower respondent acceptance and lower response rates. This is particularly pertinent in the Netherlands where response rates are low and decreasing over time [21]. With our approach of constructing a measurement instrument based on available routine public health survey data, we managed to address the issues of length and overlap and created an efficient and acceptable instrument while ensuring its validity and reliability.

The usability of the constructed instrument is not confined to the studied cities. The use of multiple datasets allowed us to replicate the measurement properties in other populations, which improved the generalisability of our findings beyond the population in which the instrument was developed. This makes it a promising instrument for other cities and countries as well.

Further strengths of our study are the large sample size, the broad representation of the study population and the intensive approach of hard-to-reach high risk groups.

A limitation of our study is that the routine public health surveys used in this study did not contain items on the dimension ‘lack of normative integration’. It has been reported previously that such items are not standardly available in (health) questionnaires [10]. Normative integration relates to the duties of social citizenship and is reflected in e.g. compliance with dominant values, social commitment and responsibility towards fellow citizens. Failure to comply with these obligations is as much a cause of social (self-)exclusion as are the rights associated with social citizenship [7]. As normative integration is considered an important theoretical dimension of social exclusion, we recommend to include in future research additional items from the validated SCP index, such as ‘giving to good causes’ and ‘sometimes doing something for one’s neighbours’ (Table 3). Although the other three dimensions were well represented in Index3, some improvements can be made. Items that could be included in the dimension ‘material deprivation’ are lack of basic goods and services and in the dimension ‘inadequate access to basic social rights’ items that refer to the actual lack of access to healthcare.

Furthermore, we were not able to assess the concurrent validity of our indices. As the study was based on secondary data, we could not examine the agreement between the indices and the SCP index in the same dataset. However, the evidence suggests that the constructed indices will be closely interrelated with the SCP index, given the similarities in content and good agreement in measurement properties between the constructed indices and the SCP index.

The main contribution of this paper is the development of a social exclusion index that can be measured reliably and validly with routine public health survey data. Until now, no generally accepted and validated instrument has been developed to measure social exclusion in health research [3], [22]–[25], even though such an instrument is considered paramount to improve our understanding of how social exclusion influences health and health inequalities [2]–[4], [22], [26], [27]. The index discussed in this article is not only relevant for the Netherlands, but may be applied in other public health surveillance systems as well, such as the Centers for Disease Control and Prevention National Health Interview Survey [28], the Health Survey for England [29] and the Italian risk factor surveillance system PASSI [30]. Once included in routine public health monitoring, large amounts of data will become available with which social exclusion can be quantified, risk groups identified and developments monitored over time. Relations with health outcomes and determinants can be assessed by combining social exclusion data with other health surveillance data. Such information is relevant from several perspectives. Social exclusion is considered an important determinant of health inequalities and offers a broader range of policy options than more simple concepts like low income and poverty [26], [31], [32]. Valid and reliable information can help policy makers to develop more effective policies to reduce health inequalities. Moreover, it can provide a baseline from which to monitor and assess the effects of policies and programmes [2], [3], [33]. Finally, the measurement of social exclusion can raise the profile and visibility of excluded groups and draw attention to the diverse causes and consequences of social exclusion [24].

This study set out to explore whether the multidimensional concept of social exclusion can be measured with the health questionnaires that are currently used in the public health surveys in the Netherlands. This question can be answered positively. We succeeded in constructing a brief measure for social exclusion with good measurement properties and high acceptability, which is suitable for use in routine public health surveys. The use of this measure in other countries and regions will enable the development of effective policies and programmes to tackle health inequalities.

## Supporting Information

Figure S1 **Centroid plots Index1: Quadrants I and II (A); Quadrants III and IV (B).** The Figures S1 A and B show the centroid plots generated by a two dimensional Overals analysis on the Amsterdam dataset. Blue are centroids of variables in the set ‘Lack of social participation’; red are centroids of variables in the set ‘Material deprivation’. The scales vary between figures.
(TIF)

Figure S2 **Centroid plots Index2: Quadrants I and II (A); Quadrants III and IV (B).** The figures S2 A and B show the centroid plots generated by a two dimensional Overals analysis on the Rotterdam/The Hague dataset. Blue are centroids of variables in the set ‘Lack of social participation’; red are centroids of variables in the set ‘Material deprivation’ and green are centroids of variables in the set ‘Limited access to basic social rights’. The scales vary between figures.
(TIF)

Figure S3 **Centroid plots Index3: Quadrants I and II (A); Quadrants III and IV (B).** The figures S3 A and B show the centroid plots generated by a two dimensional Overals analysis on the Utrecht dataset. Blue are centroids of variables in the set ‘Lack of social participation’; red are centroids of variables in the set ‘Material deprivation’ and green are centroids of variables in the set ‘Limited access to basic social rights’. The scales vary between figures.
(TIF)
