# Supplementary figures and images for: Measuring Social Exclusion in Routine Public Health Surveys: Construction of a Multidimensional Instrument
Source: PLoS One. 2014 May 30;9(5):e98680. doi: 10.1371/journal.pone.0098680 (PMC4039524; doi:10.1371/journal.pone.0098680)

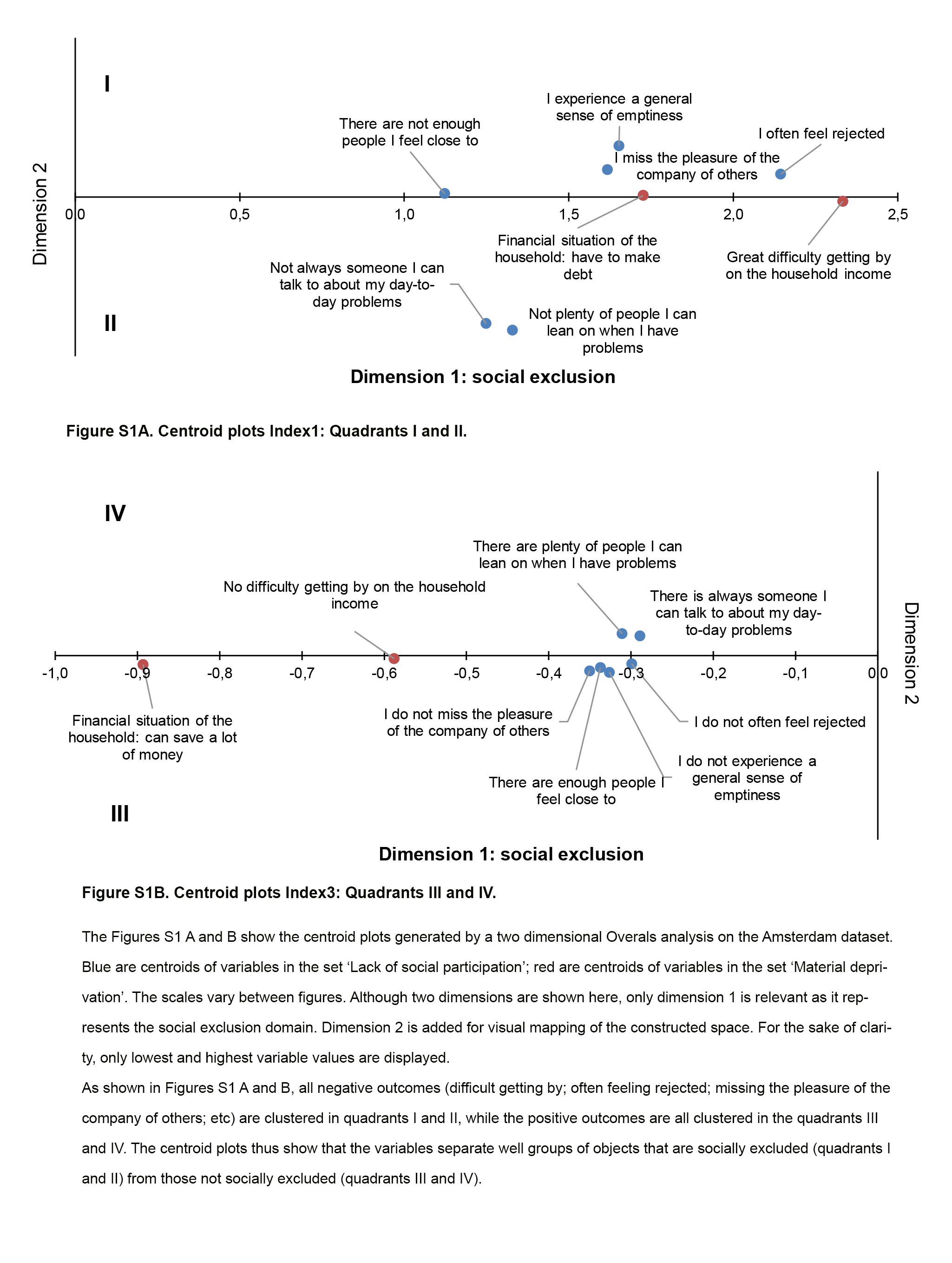

Supplement: Figure S1 — Centroid plots Index1: Quadrants I and II (A); Quadrants III and IV (B). The Figures S1 A and B show the centroid plots generated by a two dimensional Overals analysis on the Amsterdam dataset. Blue are centroids of variables in the set ‘Lack of social participation’; red are centroids of variables in the set ‘Material deprivation’. The scales vary between figures. (TIF) [file pone.0098680.s001.tif]

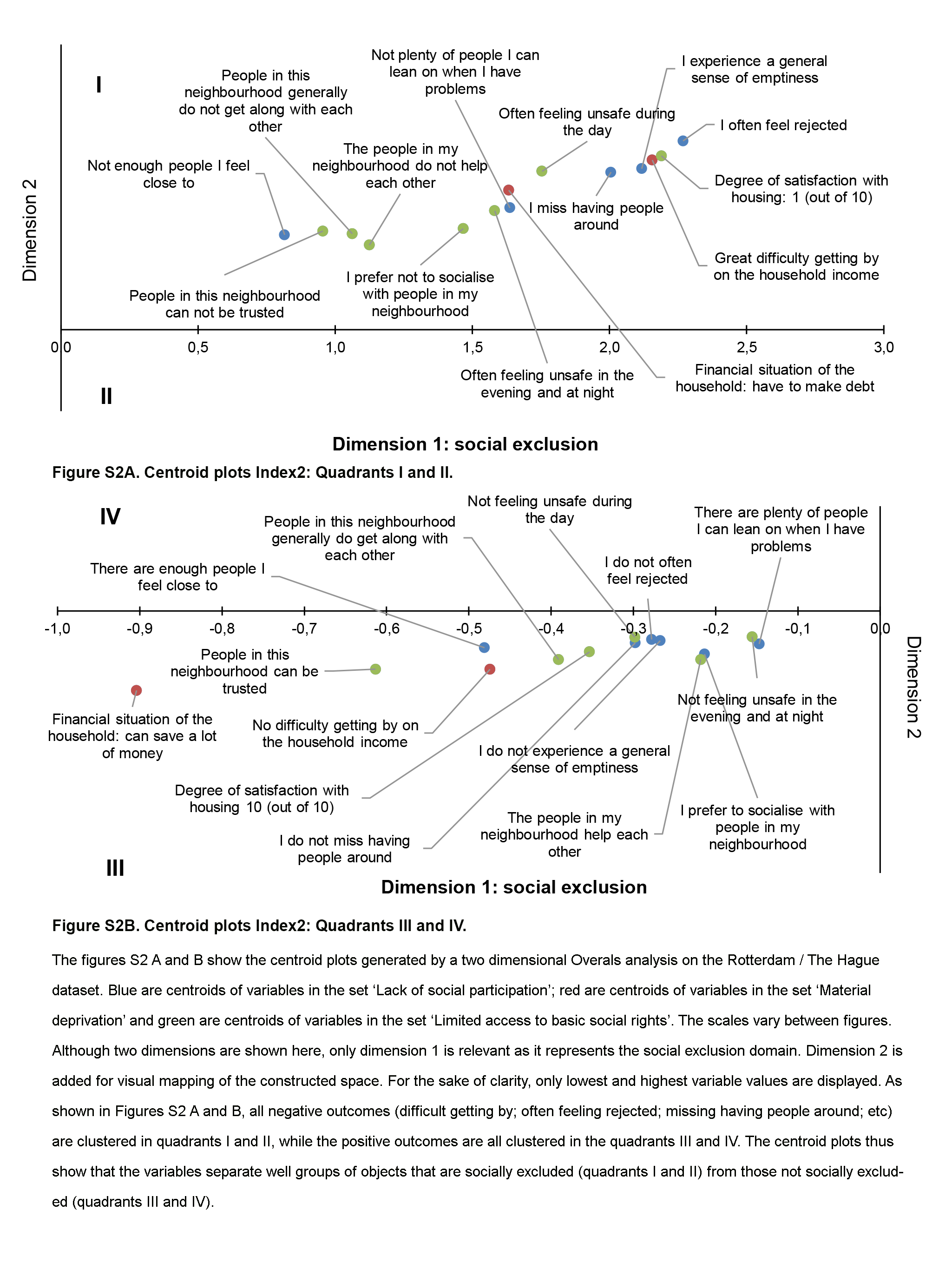

Supplement: Figure S2 — Centroid plots Index2: Quadrants I and II (A); Quadrants III and IV (B). The figures S2 A and B show the centroid plots generated by a two dimensional Overals analysis on the Rotterdam/The Hague dataset. Blue are centroids of variables in the set ‘Lack of social participation’; red are centroids of variables in the set ‘Material deprivation’ and green are centroids of variables in the set ‘Limited access to basic social rights’. The scales vary between figures. (TIF) [file pone.0098680.s002.tif]

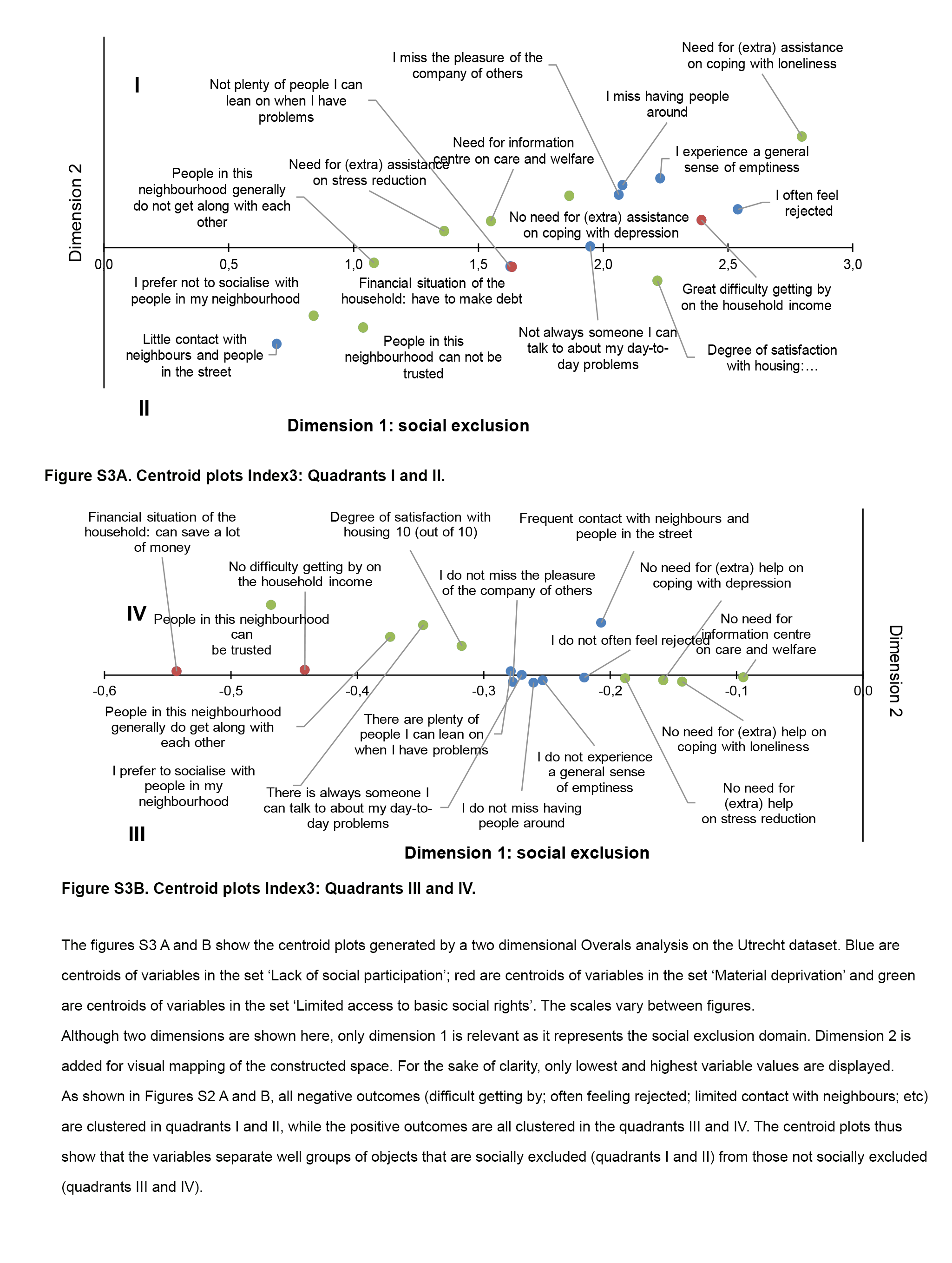

Supplement: Figure S3 — Centroid plots Index3: Quadrants I and II (A); Quadrants III and IV (B). The figures S3 A and B show the centroid plots generated by a two dimensional Overals analysis on the Utrecht dataset. Blue are centroids of variables in the set ‘Lack of social participation’; red are centroids of variables in the set ‘Material deprivation’ and green are centroids of variables in the set ‘Limited access to basic social rights’. The scales vary between figures. (TIF) [file pone.0098680.s003.tif]
